# Role of Thermography in the Diagnosis of Chronic Sinusitis

**DOI:** 10.7759/cureus.2298

**Published:** 2018-03-10

**Authors:** Raja Kalaiarasi, Chellappa Vijayakumar, Ramalingam Archana, Ramakrishnan Venkataramanan, Ranganathan Chidambaram, Sadhanandham Shrinuvasan, Ravi Prabhu

**Affiliations:** 1 Otorhinolaryngology, Sri Lakshmi Narayana Institute of Medical Science, Puducherry, India; 2 Surgery, Jawaharlal Institute of Postgraduate Medical Education and Research (JIPMER), Puducherry, India.; 3 Preventive Medicine, Jawaharlal Institute of Postgraduate Medical Education and Research (JIPMER), Puducherry, India.; 4 Otolaryngology, Sri Lakshmi Narayana Institute of Medical Science, Puducherry, India; 5 Radiology, Sri Lakshmi Narayana Institute of Medical Science, Puducherry, India; 6 General Surgery, Sri Lakshmi Narayana Institute of Medical Science, Puducherry, India

**Keywords:** temperature, sinusitis, x-rays, paranasal sinus

## Abstract

Introduction

Thermography is a form of radiography that images the skin surface temperature. Thermograms are pictorial representations of thermal maps of the entire body’s outer surface. Thermography was applied as an attempt to evaluate its usefulness in the diagnosis of chronic sinusitis (CS). Hence, this study was done to determine the diagnostic value of thermography for patients suffering from CS.

Methodology

Patients attending the Department of Otorhinolaryngology and Head and Neck Surgery over a two years' duration with symptoms suggestive of CS were included in this diagnostic evaluation study. X-ray paranasal sinuses (PNS) and nose, thermography of head and neck, and computed tomography (CT) of PNS and nose (axial and coronal sections) were performed on them. The thermograms and X-ray sinuses obtained were compared with the computed tomography of PNS findings.

Results

The study population consisted of 167 patients (75 males and 92 females) and the mean age of the study population was 38.6 years. The sensitivity and specificity of thermography of the head and neck in diagnosing frontal, ethmoidal, maxillary, and sphenoidal sinusitis were 92.59% and 68.58%, 100% and 66.32%, 70.06% and 85.88%, 99.18% and 0%, respectively. Whereas the sensitivity and specificity of the X-ray PNS and nose in diagnosing frontal, ethmoidal, maxillary, and sphenoidal sinusitis were 92.59% and 77.88%, 73.61% and 81.05%, 89.19% and 98.92%, 74.44% and 99.18%, respectively.

Conclusion

Thermography is better than X-rays in diagnosing frontal and ethmoidal sinusitis and as good as X-ray PNS and nose in diagnosing maxillary sinusitis. Thermography failed to pick up sphenoidal sinusitis. The advantages of thermography are that it is a radiation-free, non-invasive, and cost-effective method for diagnosing CS.

## Introduction

Chronic sinusitis (CS) is one of the most common diseases that affect the majority of the population. It is a group of disorders characterized by the inflammation of the mucosa of the nose and paranasal sinuses (PNS) for at least 12 consecutive weeks [1]. Many possible diagnostic methods are available to confirm the diagnosis of CS, but there is no consensus reached on the best-available, most cost-effective diagnostic method. A plain sinus radiograph is a commonly used initial diagnostic test but not of much diagnostic value. Computed tomography (CT) of paranasal sinuses (CT PNS) scans have been very helpful in diagnosing and assessing the severity of the disease. CT PNS is the gold standard test for both confirming diagnosis and assessing the response to the treatment of CS [2-3]. But there is a risk of radiation exposure, and it is an expensive investigation. Magnetic resonance imaging (MRI) scans have limited usefulness in the diagnosis of CS, as they are too sensitive in assessing the mucosal lesion and lack bony details needed for surgical treatment.

Thermography is a form of radiography that images the skin surface temperature. Thermograms are pictorial representations of thermal maps of the entire body’s outer surface. The principle behind thermography is to detect and measure variations in the heat emitted by the body in the form of infrared radiation and convert them into electrical signals [4]. The electrical impulses are fed into the computer, which analyzes the temperature and vascular changes, thereby producing high-resolution images [4-6]. The thermogram images are recorded photographically, thereby diagnosing the diseased conditions. The images of hotter areas in the body appear red and the colder areas appear blue in color. Areas of normal temperature appear green in color. In this study, thermography was applied as an attempt to evaluate its usefulness in the diagnosis of CS. This study was done to determine the diagnostic value of thermography for patients suffering from CS.

## Materials and methods

Patients attending the Department of Otorhinolaryngology and Head and Neck Surgery over a two years' duration with symptoms suggestive of CS (Rhinosinusitis Task Force's criteria) were included in this diagnostic evaluation study. Children below 15 years of age, patients with facial trauma, nasal masses, nasal polyposis, and unwilling candidates were excluded from the study. This study was approved by the Institute Ethical Committee. Detailed history and clinical examination were done followed by diagnostic nasal endoscopy (DNE) in all the patients with suspected CS. As an initial investigation, digital X-ray PNS and nose (Water’s view) was done followed by thermography of the head and neck. Before doing the thermographic imaging, patients were allowed to rest at a room temperature of 28^o^C. This was to achieve body temperature equilibrium with the surrounding ambient temperature and to fix all the variables that might influence the thermal imaging of the head and neck.

**Figure 1:**
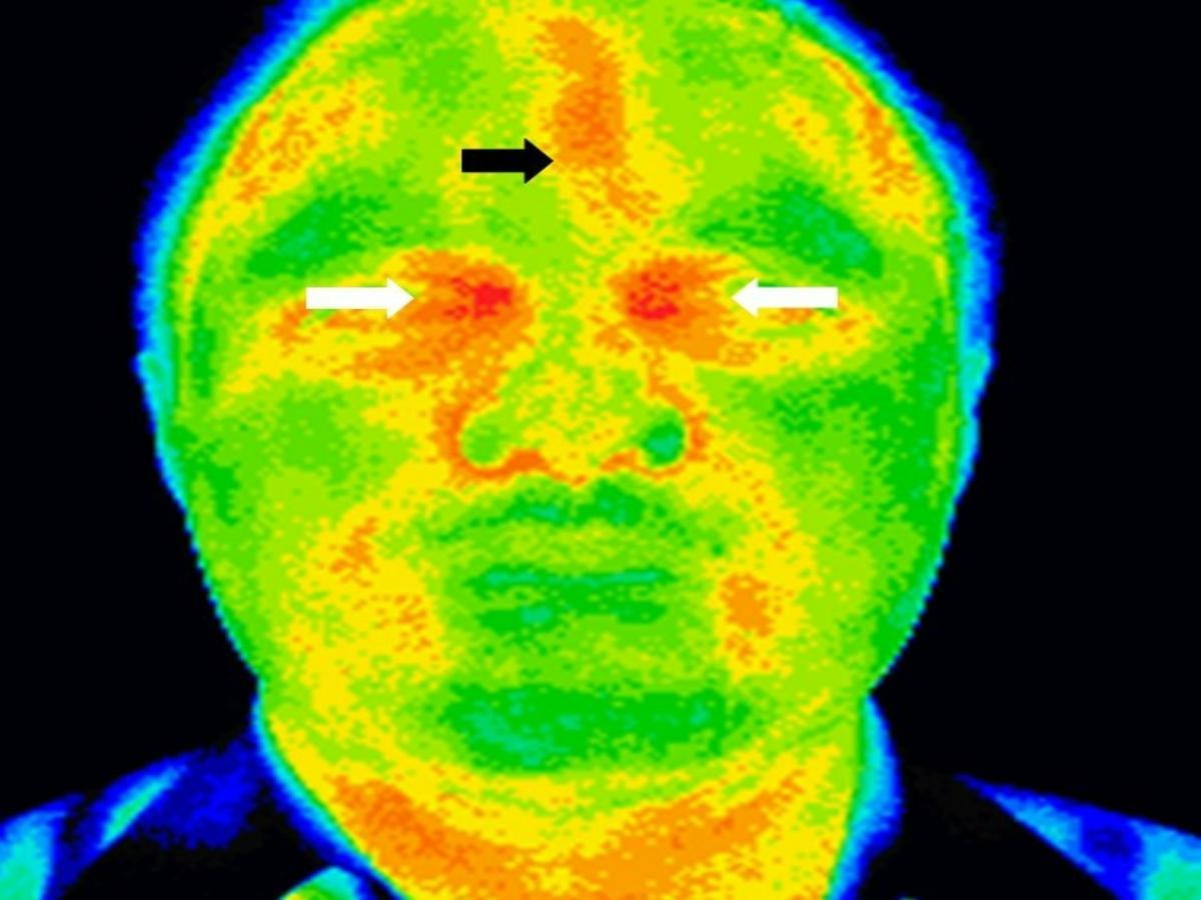
Thermography of the head and neck showing regions of hyperthermia (black arrow) in the left frontal region and bilateral ethmoid regions (white arrow) suggestive of left frontal and ethmoidal sinusitis

An infrared thermal camera was positioned one meter away from the patient’s head. Thermography of the head and neck was performed using a high-resolution digital, non-contact thermographic camera, MED2000 Elite Iris (Meditherm Inc. Digital Infrared Thermal Imaging, Cheyenne, WY, USA). The standard views - frontal view, frontal view with open mouth, right lateral, and left lateral views were taken. A thermal difference of 0.5°C between the homologous regions of the face or a difference of 0.5°C compared to the surrounding area with color change was taken as the criterion for abnormality [7]. CT PNS (axial and coronal sections) was also performed on all patients included in the study. The CT machine used in our study was the Pro-Speed Plus 4 Slice Multidetector CT machine (GE, Boston, MA, USA). The sections were taken with a slice thickness of 5 mm. In our study, CT PNS imaging with 4 mm or more mucosal thickening in the sinus cavity was taken as positive for CS [8]. Each sinus was studied separately, and data were collected. In thermography, sinuses that showed red color were taken as positive for sinusitis (Figure 1).

In a digital X-ray PNS, haziness rather than orbit was taken as positive for sinusitis. The thermography results and digital X-ray PNS results obtained were compared with the results of the CT PNS findings. Open Epi version 3 Software (Centers for Disease Control and Prevention, Atlanta, Georgia, USA) was used for analysis and the test applied was the Wilson score test.

## Results

The study population consisted of 167 patients (75 males and 92 females) and the mean age of the study population was 38.6 years. The most common symptoms reported by the patients were nasal discharge and headache, which were present in all 167 patients (100%), followed by facial fullness in 98 patients (58.7%), hyposmia in 90 patients (53.9%), and postnasal drip in 88 patients (52.7%). PNS tenderness was present in all patients. Diagnostic nasal endoscopy (DNE) was normal in 94 (56.3%) patients (Table 1).

**Table 1:** Diagnostic nasal endoscopy (DNE) findings of the study population

No.	Diagnostic Nasal Endoscopy Findings	No. of Patients (%)
1.	Normal	94 (56.3)
2.	Purulence in both middle meatus	39 (23.4)
3.	Purulence in left middle meatus	10 (6)
4.	Purulence in right middle meatus	24 (14.4)

**Figure 2:**
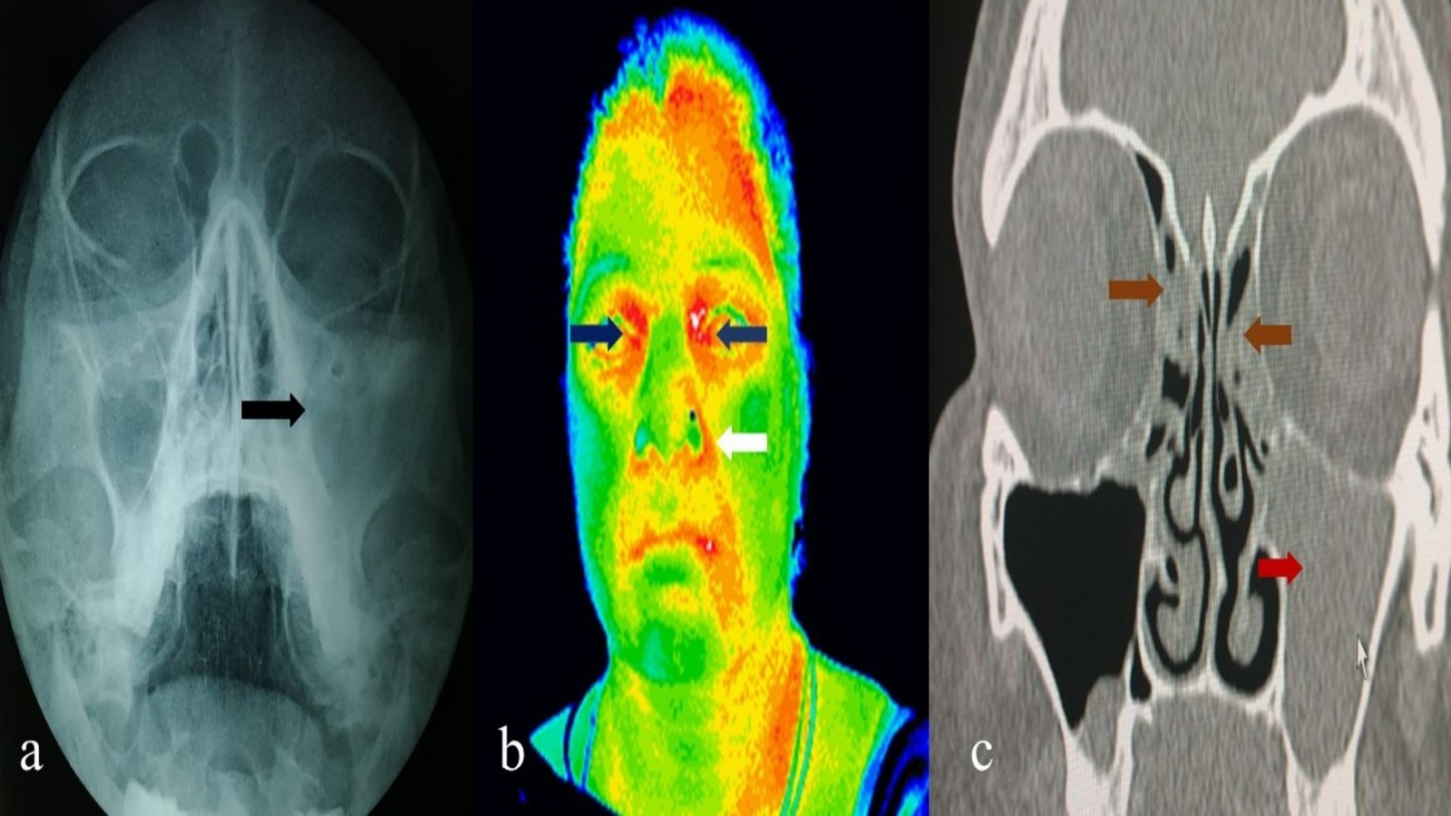
X-ray nose and paranasal sinuses (a) showing haziness in the left maxillary sinus (black arrow) and ethmoidal regions. Thermography of head and neck (b) showing regions of hyperthermia in the left nasolabial fold (white arrow) and bilateral ethmoidal regions (blue arrow) suggestive of left maxillary and bilateral ethmoidal sinusitis. Computed tomography of nose and paranasal sinuses (c) showing hyperdensity in left maxillary (red arrow) and bilateral ethmoidal sinuses (brown arrow)

Out of 167 patients, in CT PNS imaging, 55 (32.9%) patients had a right-sided frontal disease and 53 (31.7%) patients had a left frontal disease. Thermography picked up the right frontal disease in 82 (49.1%) patients and the left-sided frontal disease in 89 (53.3%) (Figure 2).

A digital X-ray showed a right-sided frontal disease in 82 patients (49.1%) and a left-sided disease in 68 patients (40.7%) (Table 2).

**Table 2:** Involvement of paranasal sinuses in computed tomography, thermography, and digital X-ray nose and paranasal sinuses No. (%); CT: computed tomography; R: right; L: left

No.	Sinuses	CT findings		Thermography findings		X-ray findings	
		Present*	Absent*	Present*	Absent*	Present*	Absent*
1.	Frontal (R)	55 (32)	112 (67)	82 (49)	85 (50)	82 (49)	85 (50)
2.	Frontal (L)	53 (31)	114 (68)	89 (53)	78 (46)	68 (40)	99 (59)
3.	Ethmoid (R)	73 (43)	94 (56)	106 (63)	61 (36)	68 (40)	99 (59)
4.	Ethmoid (L)	71 (42)	96 (57)	102 (61)	65 (38)	74 (44)	93 (55)
5.	Maxillary (R)	83 (49)	84 (50)	77 (46)	90 (53)	79 (47)	88 (52)
6.	Maxillary (L)	74 (44)	93 (55)	58 (34)	109 (65)	67 (40)	99 (59)
7.	Sphenoid (R)	47 (28)	120 (71)	167 (100)	00 (00)	154 (76)	13 (23)
8.	Sphenoid (L)	43 (25)	124 (74)	167 (100)	00 (00)	154 (76)	13 (23)

The sensitivity and specificity of thermography in diagnosing frontal, ethmoidal, maxillary and sphenoidal sinusitis were 92.59% and 68.58%, 100% and 66.32%, 70.06% and 85.88%, 99.18% and 0%, respectively, as shown in Table 3.

**Table 3:** Sensitivity, specificity, and predictive values of thermography in diagnosing chronic sinusitis % (95% confidence interval); PPV: positive predictive value; NPV: negative predictive value

No.	Sinuses	Sensitivity*	Specificity*	PPV*	NPV*
1.	Frontal	92.59 (86.06-96.2)	68.50 (62.26-74.28)	58.48 (50.99-65.6)	95.09 (90.62-97.49)
2.	Ethmoid	100.00 (97.4-100)	66.32 (59.33-72.65)	69.23 (62.66-75.11)	100.00 (97.04-100)
3.	Maxillary	70.06 (62.49-76.68)	85.88 (79.98-90.25)	81.48 (74.09-87.13)	76.38 (70.02-81.75)
4.	Sphenoid	99.18 (97.06-99.77)	00.00 (00.00-04.09)	72.89 (67.87-77.39)	00.00 (00.00-65.76)

Whereas the sensitivity and specificity of X-ray paranasal sinuses and nose in diagnosing frontal, ethmoidal, maxillary and sphenoidal sinusitis were 92.59% and 77.88%, 73.61% and 81.05%, 89.19% and 98.92%, 74.44% and 99.18%, respectively, as shown in Table 4.

**Table 4:** Sensitivity, specificity, and predictive values of X-ray paranasal sinuses and nose in diagnosing chronic sinusitis % (95% confidence interval); PPV: positive predictive value; NPV: negative predictive value

No.	Sinuses	Sensitivity*	Specificity*	PPV*	NPV*
1.	Frontal	92.59 (86.06-96.2)	77.88 (72.02-82.8)	66.67 (58.79-73.71)	95.65 (91.66-97.78)
2.	Ethmoid	73.61 (65.81-80.13)	81.05 (74.89-85.99)	74.65 (66.91-81.09)	80.21 (74.00-85.23)
3.	Maxillary	89.19 (80.09-94.42)	98.92 (94.16-99.81)	98.51 (92.02-99.74)	92.00 (85.00-95.89)
4.	Sphenoid	74.44 (65.57-80.5)	99.18 (97.06-99.77)	87.44 (81.12-89.29)	95.21 (92.36-97.03)

## Discussion

Inflammatory processes are characterized by high temperature, which is the basis for the use of thermography in medical practice. Thermography has principle application in screening and diagnosing breast diseases and in arteriovenous diseases [4-6]. In both fields, thermography has been used as the screening method and to assess the treatment response [9-10]. The use of thermography in the diagnosis of CS has been seldom reported in the literature. In the literature, there was a study concerning its use in PNS diseases [4,11]. Thermography provides information regarding the functional aspects and does not give information about the morphological characteristics of the sinuses.

Various studies reported the sensitivity and specificity of the X-ray PNS in diagnosing sinusitis, which ranges from 41%-95% and 30%-85%, respectively [12-15]. But the diagnostic ability of the X-ray PNS with respect to individual sinuses was not studied. In this study, we found that thermography is better than the X-ray PNS in diagnosing frontal and ethmoidal sinusitis. This is because of the location of the sinuses in comparison with the skin surface. Thermography is as good as the X-ray PNS in diagnosing maxillary sinusitis. However, thermography failed to pick up sphenoidal sinusitis. This is because of the deeper location of the sphenoid sinus with respect to the skin surface. Both the X-ray PNS and thermography are not very reliable diagnostic methods to identify sphenoidal sinusitis.

The role of thermography is only supplementary to other techniques, as it is a test of physiology that alone is not sufficient for physicians to make or confirm a diagnosis. The limitations associated with the wide application of infrared thermography in research include the expertise required to interpret the results and the need for doing the test in a highly controlled environment. The specificity of this modality is less, but it can be compensated by correlating with a clinical examination, which can enhance its diagnosing ability. The limitation of the study was the relatively smaller sample size of the study population.

The advantages of thermography are early detection and its radiation-free, non-invasive, painless, and cost-effective modality. Correctly done thermography provides helpful information about sinus diseases without the risk of radiation exposure. Thermography does not supersede CT and X-ray scanning or other necessary methods of diagnosis for CRS. Infrared thermography is a good supplemental test and further large-scale research is necessary to determine whether it can be used as a diagnostic tool for diagnosing CRS.

## Conclusions

Thermal imaging is a harmless imaging method that can be easily implemented in routine clinical practice. We conclude that thermography is a useful supplementary investigation in gaining information regarding sinus diseases. However, it should not be considered as a standalone diagnostic tool in itself but interpreted in conjunction with other clinical clues in the patient’s history and examination findings.
